# Detection of *Haemophilus ducreyi* from environmental and animal samples in Cameroon

**DOI:** 10.1371/journal.pntd.0013091

**Published:** 2025-05-08

**Authors:** Philippe Ndzomo, Serges Tchatchouang, Onana Boyomo, Tania Crucitti, Michael Marks, Sara Eyangoh

**Affiliations:** 1 Scientific Direction, Centre Pasteur du Cameroun, Yaounde, Cameroon; 2 Department of Microbiology, Faculty of Science, University of Yaounde 1, Yaounde, Cameroon; 3 Experimental Bacteriology Unit, Institut Pasteur de Madagascar, Antananarivo, Madagascar; 4 Clinical Research Department, Faculty of Infectious and Tropical Diseases, London School of Hygiene and Tropical Medicine, London, United Kingdom; 5 Hospital for Tropical Diseases, University College London Hospital, London, United Kingdom; 6 Division of Infection and Immunity, University College London, London, United Kingdom; Jawaharlal Nehru Medical College and Hospital: Jawaharlal Nehru Medical College, INDIA

## Abstract

**Background:**

Children in parts of Africa, the South Pacific, and Southeast Asia frequently develop cutaneous ulcers caused by two bacteria: *Haemophilus ducreyi* (HD) and *Treponema pallidum* subspecies *pertenue* (causative agent of yaws). The World Health Organization (WHO) aims to eradicate yaws using mass administration of azithromycin. This also leads to a temporary decrease in ulcers caused by HD followed by a rebound suggesting an ongoing reservoir of infection. The aim of this study was to investigate whether HD could spread through the environment or animals.

**Methods:**

Alongside detection of human cases of cutaneous ulcers from villages in Cameroon, we additionally collected samples from animals (dogs, cats, flies), fomites (bedsheets, clothing, benches, doors), and water sources (marigots and lakes). DNA was extracted and tested for HD and *T. pallidum* using two specific qPCR assays.

**Results:**

HD was not detected in any of the environmental samples but it was on both clothing (13.3%) and in flies (27%). Flies also tested positive for *T. pallidum,* but at a lower rate (2.6%).

**Conclusions:**

These results suggest that flies and some fomites may contribute to the transmission of HD. Future research should focus on determining whether either of these are capable of carrying live bacteria that can cause onward transmission.

## Author summary

Cutaneous ulcers are a significant public health concern, particularly among children in tropical regions of the Pacific, Southeast Asia, West and Central Africa. These lesions are primarily caused by HD, the etiological agent of chancroid, and *Treponema pallidum* subspecies *pertenue*, responsible for yaws. To eliminate these infections by 2030, WHO advocates mass drug administration (MDA) with azithromycin, a more effective and less invasive alternative to benzathine penicillin. However, HD infections persist in endemic areas despite these efforts. In this study, we explored the potential role of environmental and animal reservoirs in sustaining HD transmission. Our findings confirm the presence of HD in flies and on clothing, suggesting that these may act as mechanical and passive vectors, respectively, contributing to the persistence of ulcers even after antibiotic treatment. These results underscore the need for complementary strategies, including improved hygiene measures, to effectively achieve eradication.

## Introduction

Non-genital skin ulcers remain a significant public health problem in many tropical regions. In many parts of the world, *Treponema pallidum* subspecies *pertenue* has historically been considered the primary causative agent of these ulcers. However studies in several countries have more recently demonstrated that HD is a major cause of cutaneous ulcers [1,2]. This recognition has raised new questions regarding the transmission and persistence of HD in these settings [3].

WHO recommends mass drug administration of azithromycin to eradicate yaws, and this antibiotic has also proven highly effective in treating HD ulcers [4]. These campaigns temporarily reduce the burden of HD ulcers, however, the rebound and persistence of lesions post-interventions suggests that alternative transmission routes may be involved [3,5,6]. One hypothesis is the existence of an environmental or animal reservoir that supports the ongoing spread of the pathogen, even after treatment. Contaminated surfaces (fomites) and certain animals might serve as indirect vectors, contributing to the recurrence of the disease.

A previous study in Papua New Guinea (PNG) detected HD DNA in fomites, providing initial evidence that non-human sources may play a role in the persistence of this pathogen [3]. Building on this finding, further investigations are needed to clarify the potential role of environmental or animal reservoirs in HD transmission. The detection of HD in non-human samples is therefore crucial for improving our understanding of its transmission dynamics. By expanding on existing evidence, this study aims to explore whether additional reservoirs contribute to HD persistence and, ultimately, to inform more effective control strategies. If an environmental or animal reservoir is confirmed, complementary interventions beyond mass drug administration may be necessary to break the transmission cycle.

## Methodology

We analysed samples collected in 2021 as part of investigation into the prevalence and risk factors associated with HD cutaneous ulcers in yaws-endemic health districts of Cameroon [7]. We selected the districts of Bankim, Doumé, and Yokadouma from among the fourteen districts investigated in our previous study, based on their high HD detection rates. This selection aimed to increase the likelihood of detecting a potential human-animal-environment transmission chain. These sites also offered suitable ecological conditions, including the presence of water bodies and domestic animals, facilitating environmental and animal sampling. In these sites, we identified households and classrooms where at least one individual presented with cutaneous ulcers considered to be clinically consistent with yaws.

To investigate potential environmental sources, 500 ml water samples were collected from community water points, including backwaters and lakes. In addition, swab samples were collected from various inanimate surfaces such as bed linens, clothes, doors, and desk benches by rolling the moistened polyester tipped swabs with polystyrene handle (Puritan sterile swabs, Cat #25–806 1PD, Puritan Medical Products Guilborg, UK) over the entire visible area.

To assess animal involvement, a set of 20 flies was collected from verandas and surrounding areas near patients’ households or classrooms. Flies were pooled in falcon tubes containing 5 ml of a custom-made lysis buffer (10 mM Tris, pH 8.0, 0.1 M EDTA, pH 8.0, 0.5% SDS). Swabs were also taken from domestic animals, primarily dogs and cats. Two swabs were taken per animal (one from the inner mouth surface and one from the fur) and pooled in 500 µL of the custom-made lysis buffer.

All samples were kept refrigerated at 2–8°C until they could be transported to the Centre Pasteur du Cameroun (CPC), where they were stored at −20°C till analysis.

Genomic DNA extraction from water samples was performed using the DNeasy PowerWater kit (Qiagen, Germany), following the manufacturer’s instructions, with an initial filtration step using Whatman paper. For fly samples, analysis of the lysis buffer and the whole fly was carried out separately. For the lysis buffer, DNA extraction was performed directly from a 200 µL volume. For the whole flies a grinding step was performed on a mortar using liquid nitrogen and the resulting powder was used for DNA extraction using the DNeasy Blood & Tissue kit (Qiagen, Germany) according to the manufacturer’s recommendations. The same kit was used for DNA extraction from swab samples collected from domestic animals, bed linens, doors, clothes, and desk benches from an initial volume of 200 µL.

HD DNA detection was achieved by targeting the V8 region of the 16S ribosomal RNA gene. Primers, probe and qPCR conditions were published earlier [7]. *T. pallidum* was detected by amplifying the *pol A* gene using a previously described protocol [8]. Before implementing this qPCR assay, we performed all validation, verification, and quality control steps to ensure its reliability for HD detection. These included assessments of sensitivity, specificity, primer specificity, repeatability, reproducibility, and PCR efficiency. Positive control was included in each run to validate performance, with consistent Ct values confirming assay robustness and minimizing inter-run variability. Negative control was also added in each run to validate the manipulation.

## Results

A total of 188 non-human samples were collected across three health districts. For environmental sampling, 33 water samples were collected, of which 26 (78.8%) were from backwaters and 7 (21.2%) from lakes. In addition, 89 fomites samples were collected, with 30 (33.7%) from clothes, 9 (10.1%) from bed linens, and 50 (56.2%) from surfaces including school-benches and doors. A total of 66 samples were gathered from animals including 40 pools of flies, 19 samples from dogs, and 7 from cats.

No HD was detected in the 33 water samples regardless of whether they were collected from backwaters or lakes. Among the 89 fomites sample, HD was identified in four of the 30 clothes samples (13.3%) while no detection was recorded in the bed linens or other surfaces giving an overall positivity rate of 4.5%. Amongst samples collected from animals, HD was detected in six (15%) fly samples from lysis buffer and 11 (27.5%) from whole flies. No other animal sample tested positive. Only a single environmental sample and a single fly sample tested positive for TP (Table 1).

**Table 1:** Detection of HD in non-human samples.

Type of samples	Subtype	Total number n	HD DNA detected n (%)	TP DNA detected n (%)
**Water**	Backwaters	26	0 (0)	0 (0)
	Lakes	7	0 (0)	0 (0)
**Animals**	Dogs	19	0 (0)	0 (0)
	Cats	7	0 (0)	0 (0)
	Fly (lysis buffer)	40	6 (15)	1 (2.5)
	Fly (whole fly)	40	11 (27.5)	0 (0)
**Surfaces**	Clothes	30	4 (13.3)	1 (3.3)
	Bedsheet	9	0 (0)	0 (0)
	Benches	32	0 (0)	0 (0)
	Doors	18	0 (0)	0 (0)

**Fig 1:**
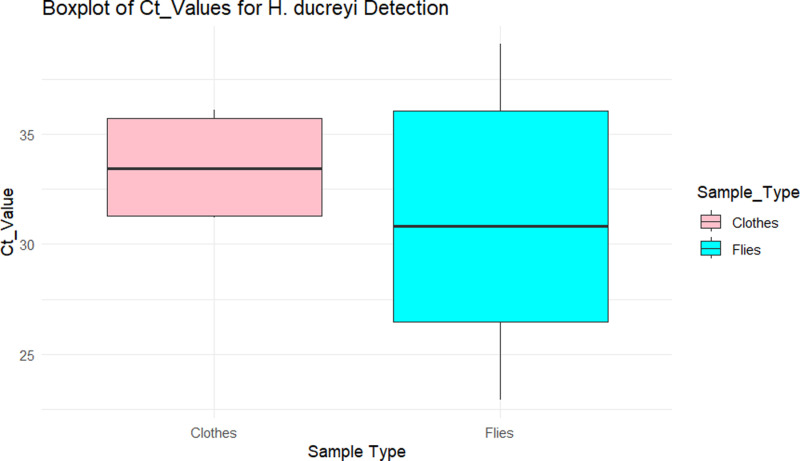
Boxplot comparing Ct values for HD in flies and clothes. The plot was generated using R software V.4.3.1.

The cycle threshold (Ct) values for HD positive samples indicated variations in pathogen load across sample types. Flies exhibited a mean Ct of 31.25 ± 5.51 (range: 22.9–39.1), suggesting relative higher pathogen load compared to clothes, which showed a mean Ct of 33.55 ± 2.66 (range: 31.2–36.1) (Fig 1).

## Discussion

The persistent transmission of HD after community wide treatment remains a significant public health challenge and suggests that current strategies are inadequate to interrupt transmission. In this study we found HD on both fomites and flies suggesting they could potentially play a role as reservoirs for persistence and potentially as routes of transmission.

The highest detection rate of HD was in flies. The presence of DNA in 15% of lysis buffers and 27.5% of whole flies supports the hypothesis that these insects may be able to act as mechanical vectors. This phenomenon is well-documented for several bacterial infections, where flies serve as passive carriers of skin pathogens, especially in tropical regions [9,10]. Given their frequent contact with bodily fluids, including exudates from skin ulcers, flies could easily spread the pathogen as they move from one host to another. Evidences of transmission of viable HD by *Musca domestica*, a common housefly has been demonstrated in PNG using a green fluorescent protein (GFP)-tagged strain [11]. The wide Ct range values observed in fly samples may be attributed to the pooling method used during testing; variability in the proportion of HD- positive flies within each pool likely influenced the overall bacterial load detected. Additionally, individual flies may carry varying bacterial loads, further contributing to the observed range. The relatively high bacterial load in the analyzed samples, as reflected by the low Ct values support a plausible indirect transmission pathway for HD-associated skin ulcers, analogous to the transmission mechanisms observed for other pathogens such as *Chlamydia trachomatis* [12,13], *Staphylococcus aureus* [14] or *T. pallidum* [15]. The combination of close human interaction, inadequate hygiene, and high fly density in endemic regions likely enhances the potential for the spread of HD via flies.

The detection of HD in 13.3% of clothing samples indicates that garments could potentially serve as reservoirs or passive vectors, particularly when in contact with infectious lesions. Porous materials like clothing provide a better condition for microbial adherence compared to non-porous surfaces like, tables, and doors, where no pathogens were detected. These findings are consistent with other studies on skin infections, which have shown that fabrics and clothing, especially when damp or in prolonged contact with the skin, can act as indirect transmission vectors [16]. Interestingly, HD DNA was not detected in bedclothing, despite the comparable porosity of fabrics like cotton. This discrepancy likely reflects differences in exposure and contamination dynamics rather than material properties alone. Clothes are typically in direct and sustained contact with infected lesions compared to bedclothing, increasing the chance of bacterial transfer. Further research is needed to assess bacterial persistence across fabric types and under varying conditions. While fomites play a secondary role compared to direct contact, they are still significant in low-hygiene environments where person-to-person transmission is more likely. In this context, the higher HD DNA loads observed in flies compared to fomites could be attributed to their biological and behavioral characteristics. Unlike passive surfaces, flies actively feed on ulcer exudates, which contain high concentrations of HD, and can retain bacterial DNA both in their gut and on their body surfaces. This repeated and direct interaction with infectious material likely accounts for the higher detectable DNA levels. In contrast, bacterial survival on fomites is subject to environmental stressors such as desiccation and UV exposure, which can reduce DNA persistence over time. These findings reinforce the need for integrated control measures, combining improved hygiene, regular laundering of clothing and bedding, and potential vector control strategies [17].

The absence of HD detection in water samples from both backwaters and lakes suggests that these environments are not conducive to the survival of this pathogen. HD is a fastidious bacterium that typically survives only under specific conditions, such as controlled humidity and temperature, like those found on human skin [18]. Our data aligns with previous studies showing that the pathogen is primarily transmitted through direct contact with skin lesions and is unlikely to persist in aquatic environments [19]. Overall, the data would appear to rule out water as a transmission canal in yaws-endemic regions.

Although HD was detected frequently in fomites and flies, *T. pallidum* was rarely detected (1.1% in fomites and 2.5% in flies). This may be due to differences in the biology and transmission modes of the two pathogens. While HD is often found in open skin lesions, facilitating its adherence to fomites and insects, *T. pallidum* is generally more difficult to detect outside human hosts [20]. This variation in detection rates could reflect differences in the environmental persistence mechanisms of these pathogens or differences in their interactions with fomites and biological vectors.

Our study has a number of limitations. Whilst we sampled a wide range of environmental and animal reservoirs, we cannot preclude the possibility that there are other reservoirs which we did not assess. Secondly, we relied on DNA detection rather than bacterial culture. As such our results do not provide definitive proof of the carriage of viable bacteria on either flies or fomites. Culture of HD is technically challenging and culture of animal and fomite samples would be especially difficult due to the likelihood of contamination of culture-based methods. Further studies to confirm the viability of HD in these contexts would be of value. Finally, we were not able to quantify the relative contribution of direct and indirect transmission routes in ongoing transmission of HD.

Overall, these results suggest that fomites and flies may contribute to HD transmission in endemic areas. Our findings on HD DNA detection in flies build on previous studies by further exploring the potential role of flies as mechanical vectors in the HD transmission cycle. While earlier research has identified HD in environmental samples such as fomites, evidence of its presence in flies has been limited. By detecting HD DNA in flies, our study provides additional support for the hypothesis that insect vectors may contribute to the persistence and spread of the pathogen, particularly in regions with high transmission. This complements previous work by expanding the range of non-human reservoirs under investigation and highlights the importance of considering insect vectors in HD transmission dynamics. Consequently, it may be necessary for control strategies to consider extending beyond managing human cases to include vector control measures and improved hygiene conditions. Consequently, control strategies may need to extend beyond managing human cases to include vector control measures and improved hygiene conditions. Given the potential role of environmental reservoirs in sustaining transmission, reinforcing individual hygiene practices should be emphasized as a key public health measure. Ensuring access to clean water, promoting regular handwashing with soap, and maintaining clean skin could significantly reduce the risk of HD infection, particularly in endemic regions where poor hygiene and high fly density facilitate disease spread. Additionally, considering the localized nature of HD-associated ulcers, topical antibiotics could serve as viable treatment options. Future trials should explore their efficacy as alternatives or adjuncts to systemic antibiotics, especially in settings where mass drug administration is impractical or reinfection rates are high. However, any treatment strategy should be integrated with hygiene and sanitation measures to effectively disrupt the transmission cycle and prevent recurrence.

## Supporting information

S1 File Ct values of positive fly and clothe samples for *H. ducreyi* detection. (DOCX)
