# Supplementary material for: Detection of Haemophilus ducreyi from environmental and animal samples in Cameroon
Source: PLoS Negl Trop Dis. 2025 May 8;19(5):e0013091. doi: 10.1371/journal.pntd.0013091 (PMC12133173; doi:10.1371/journal.pntd.0013091)
Supplement: S1 File — (DOCX) [file pntd.0013091.s001.docx]

**Ct values of positive fly and clothe samples for *H. ducreyi* detection**

| Sample type | ID | Ct Value |
| --- | --- | --- |
| Flies | FBA1 | 36.3 |
|  | FBA3 | 26.4 |
|  | FBA4 | 36.3 |
|  | FBA9 | 26 |
|  | FBA17 | 30.8 |
|  | FBA22 | 28.2 |
|  | FBA25 | 35.4 |
|  | FDO26 | 35.8 |
|  | FDO29 | 22.9 |
|  | FDO33 | 26.5 |
|  | FDO37 | 39.1 |
| Clothes | HYO5 | 35.6 |
|  | HBA13 | 31.2 |
|  | HBA18 | 36.1 |
|  | HDO20 | 31.3 |
